# Corneal Bee Sting Controlled with Early Surgical Intervention and Systemic High-Dose Steroid Therapy

**DOI:** 10.1155/2014/140626

**Published:** 2014-12-16

**Authors:** Jung-Hoon Kim, Moosang Kim, Seung-Jun Lee, Sang Beom Han, Joon Young Hyon

**Affiliations:** ^1^Department of Ophthalmology, Kangwon National University Hospital, Kangwon National University Graduate School of Medicine, 156 Baengnyeong-ro, Chuncheon, Gangwon 200-722, Republic of Korea; ^2^Department of Ophthalmology, Seoul National University Bundang Hospital, Seoul National University College of Medicine, Seongnam, Gyeonggi 463-707, Republic of Korea

## Abstract

A 34-year-old Asian woman presented with painful corneal bee sting. Examinations revealed severe corneal swelling with stinger stuck in deep stroma and endothelial cell loss. She was treated with early surgery including stinger removal and anterior chamber irrigation combined with systemic high-dose steroid therapy. Vision and corneal clarity was recovered in 5 days and no additional corneal endothelial damage was observed. This report suggests that early surgical intervention and high-dose steroid therapy appear to be a useful option in the treatment of corneal bee sting.

## 1. Introduction

Corneal bee sting is a rare condition that can lead to potentially devastating complications including keratitis, corneal opacity, uveitis, iris atrophy, glaucoma, cataract, lens subluxation, bullous keratopathy, optic neuritis, and retinopathy [1–3]. Treatment is usually aimed at prevention of secondary infection and minimizing the toxic and immunologic response. However, due to the rarity of the injury, there is no established clinical guideline for the management of the patients [3]. We recently experienced a case of corneal bee sting that was controlled with early surgical intervention and systemic high-dose steroid therapy and thus herein report the case.

## 2. Case Report

**Figure 1:**
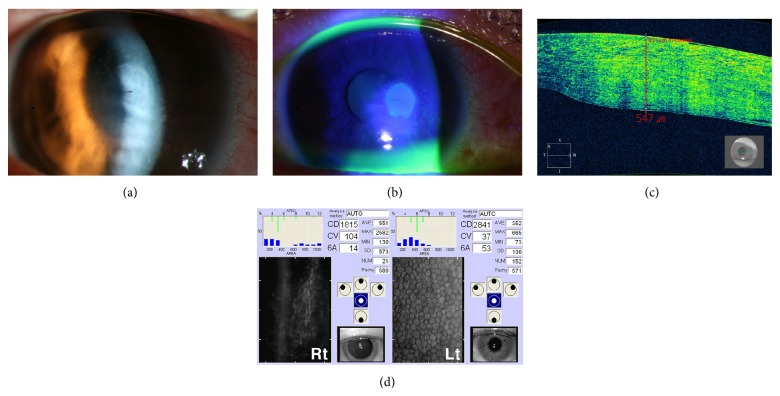
(a) Anterior segment photography depicting severe corneal edema with stinger in deep stroma. (b) Anterior segment photography showing round epithelial defect. (c) Anterior segment optical coherence tomography demonstrating severe corneal swelling and markedly increased corneal thickness (931 *μ*m) in the affected area. (d) Specular microscopy revealing substantially decreased endothelial cell density in the right eye compared to the left eye.

A 34-year-old Asian woman was referred to us with painful corneal bee sting to her right eye (OD). Past medical history was unremarkable. Her best-corrected visual acuity (BCVA) was 20/30 OD. Anterior segment examination showed conjunctival hyperemia, round epithelial defect with severe stromal edema. Bee stinger was embedded in deep stroma located at 3 o'clock in the paraxial area, 1.5 mm from the corneal center. Grade 1 anterior chamber reaction was observed. Anterior segment optical coherence tomography (AS-OCT; Carl Zeiss Meditec, Dublin, CA, USA) revealed marked corneal swelling. Specular microscopy (SP-9000; Konan medical, Tokyo, Japan) showed substantially decreased endothelial cell density (ECD) OD compared to the fellow eye (1815 versus 2841 mm^2^) (Figure 1). Funduscopic examination revealed clear vitreous and normal optic disc and retina. She received topical 0.6% besifloxacin and 1% prednisolone acetate every 2 h, respectively, and intravenous moxifloxacin 400 mg/day and oral methylprednisolone 60 mg/day. She underwent removal of the stinger with debridement of adjacent necrotic tissue using 27G needle and irrigation of the wound and irrigation of the anterior chamber with balanced salt solution (BSS).

**Figure 2:**
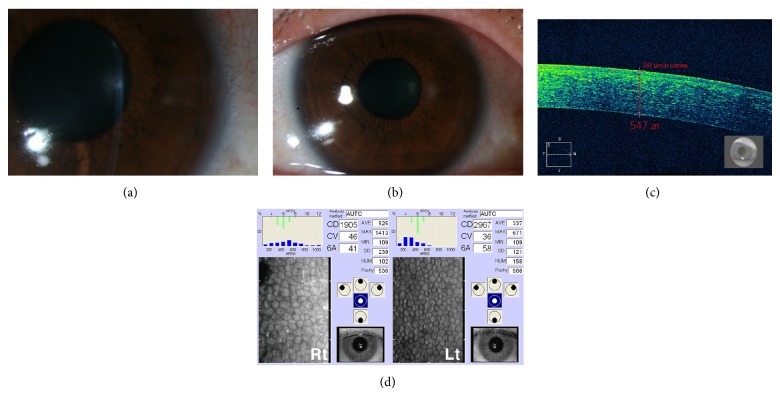
(a) Anterior segment photography showing resolution of corneal edema with small opacity around the site of bee sting. (b) Anterior segment photography showing the restoration of corneal clarity except small opacity around the site of injury. (c) Anterior segment optical coherence tomography demonstrating complete resolution of corneal swelling and normal corneal thickness (547 *μ*m). (d) Specular microscopy revealing no additional loss in endothelial cell density in the right eye, although decreased endothelial cell density in the right eye.

Five days later, her BCVA improved to 20/20 OD. Stromal edema resolved completely, although small opacity at the site of bee sting remained. AS-OCT demonstrated complete improvement of corneal swelling. Although decreased ECD was still observed, no evidence of further damage to the corneal endothelium was detected (Figure 2). Anterior chamber reaction was absent. She was observed with tapering of systemic and topical steroid. One month later, her BCVA was 20/20 OD. There was no corneal edema and no further decrease in ECD.

## 3. Discussion

Bee venom is a complex toxin that compromises various components including melittin, apamin, adolapin, phospholipase A2, hyaluronidase, and histamine. Corneal sting by bee or wasp can lead to toxic or immunologic ocular inflammation provoked by the complex venom compounds and often leaves vision-threatening sequelae including corneal opacity, bullous keratopathy, optic neuropathy, and even phthisis [1, 2, 4].

To prevent the serious complications, treatment has been mainly aimed at the control of the inflammatory response. Although there is no uniform management algorithm [3], anecdotal case reports suggested medical treatment including topical steroids, antihistamines, and cycloplegics [1, 2, 4–6]. Systemic use of steroid has also been reported [2, 5]. Although there is a controversy regarding the necessity of stinger removal, it is generally accepted that immediate removal of the stinger is required in cases associated with corneal edema and infiltration [3]. Although some cases improved without leaving serious sequelae with these treatment modalities [3, 6], cases refractory to the treatment and resulting in serious visual impairment have been reported [1, 2, 4].

In the present case, we chose early surgical intervention because of severe corneal swelling and ECD decrease (Figure 1), which suggest massive toxic or immune inflammation and endothelial cell decompensation, respectively. Because direct removal of the stinger was impossible as it was stuck in deep stroma, we removed it with 27G needle under microscope. Debridement of necrotic stromal tissue and copious irrigation of the wound was also performed to eliminate the venom completely. Irrigation of anterior chamber was done to remove the venom in the intracameral space, as substantial ECD loss immediately after the trauma suggested the activity of venom in the anterior chamber and further damage to endothelium could result in permanent corneal endothelial decompensation. As intracameral venom can lead to complications including cataract, glaucoma, and bullous keratopathy, elimination of the venom in the intracameral space has been advocated [2, 4]. A previous report that corneal endothelial decompensation developed even 1 year after bee sting also supports the necessity of thorough removal of the venom in the stroma and intracameral space [5].

We also used high dose systemic steroid to suppress the active inflammation, as suggested in previous reports [2, 5]. With the intensive initial treatment, we obtained visual recovery and restoration of corneal clarity in 5 days, which is substantially short period compared to 2 weeks–3 months in other case reports [4–6]. Additional damage to corneal endothelium was also prevented with the treatment.

We believe the intensive treatment including early surgical intervention and high-dose steroid therapy has advantage as follows. (1) Sight-threatening serious sequelae can be prevented by eliminating the venom and blocking the inflammatory response in early stage. (2) Rapid visual recovery enables early return to work and daily life and thus can promote quality of life of the patients. However, corneal bee sting can sometimes be associated with secondary infection [7, 8], and high-dose systemic steroid can be disadvantageous in these situations. Thus, we believe attention should be paid to the risk of secondary infection when administering high-dose steroid. In the present case, we used systemic and topical antibiotics for prevention of the secondary infection.

Meanwhile, favorable outcome in this case might also be due to the species of the hymenoptera responsible for the injury, considering that bee sting tends to have better prognosis compared to wasp sting [1]. Variation in the composition of venom components might result in the difference in the degrees of toxic and immunologic response [1]. However, we believe that early intensive treatment should still be considered even in bee sting as cases of serious visual impairment following bee sting have also been reported [4, 5].

In conclusion, this report suggests that early surgical intervention including stinger removal and anterior chamber irrigation combined with systemic high-dose steroid therapy may be an effective treatment option for corneal bee sting.
